# Hyperinsulinemic State and Hypokalemic Quadriparesis in Tropical Fever: Is There a Link?

**DOI:** 10.7759/cureus.57739

**Published:** 2024-04-06

**Authors:** Amitabha Saha, Arjun Talapatra, Sushmita Basu, Souvik Sarkar, Sourav Sarkar

**Affiliations:** 1 Critical Care Medicine, AMRI Hospitals, Kolkata, IND; 2 Respiratory Medicine, Datta Meghe Institue of Higher Education and Research, Wardha, IND; 3 Anesthesiology, Rama Medical College, Hospital & Research Centre, Kanpur, IND

**Keywords:** tropical fever, quadriparesis associated with dengue, hypokalemia, hyperinsulinemia, dengue fever/complications

## Abstract

It is rare for quadriparesis to manifest as a symptom of tropical illnesses. With a history of only one fever episode one week prior, our patient, a 48-year-old male with obesity and prediabetes, who was also known to have ankylosing spondylitis, presented with acute onset flaccid quadriparesis. He did not exhibit any additional symptoms of dengue, such as bleeding tendencies, petechial rashes, thrombocytopenia, or febrile episodes. Upon examination, it was discovered that he had extremely low serum potassium levels and was dengue non-specific antigen 1 (NS1) positive. His hyperinsulinemia, as seen by elevated C peptide levels, most likely caused a transcellular shift that was then triggered by the dengue infection, leading to hypokalemic paralysis.

## Introduction

In tropical regions, dengue fever is a common arboviral disease spread by mosquitoes and is commonly manifested as fever, severe headache, joint aches, and malaise. It can lead to a number of complications, including the more common dengue shock syndrome and dengue hemorrhagic fever. The central nervous system manifestations of dengue fever are well recognized and include encephalitis, opsoclonus-myoclonus, encephalomyelitis, and Guillain-Barre syndrome. However, dengue fever-related hypokalemic periodic paralysis is a very uncommon condition that has only been documented in a small number of case reports [1-3]. There are published case reports of hypokalemia induced by dengue fever leading to multiple neurological complications. Insulin resistance is an impaired biological response to insulin stimulation of target tissues. A high level of insulin resistance in the prediabetic state has been shown to induce multiple channelopathies [4]. Hypokalemia caused by the dengue virus is postulated to occur due to an increased intracellular influx of potassium [1]. However, the association of insulin resistance with dengue-induced hypokalemia has not been studied adequately. 

## Case presentation

A 48-year-old man arrived at the emergency room with weakness in all four limbs. He developed one bout of fever seven days ago and three to four episodes of loose stools which resolved after one day but was followed by a cough and cold lasting for two days. He appeared to be recovering but started experiencing acute upper limb weakness one day ago before admission which then spread to the lower limbs and he presented to us with quadriparesis. There was no prior history of respiratory distress, unconsciousness, or bleeding symptoms. Bowel and bladder abnormalities were absent. He was taking etanercept for ankylosing spondylosis, a condition for which he previously received medical attention. He also had prediabetes and had not yet begun using insulin or oral hypoglycemic medications. There was no recent change in the patient's medication history, nor was there a history of using any other medications.

Upon assessment, he had a Glasgow Coma Scale (GCS) of 15/15 and was completely cognizant and his vitals were within normal limits. With a body mass index of 32.2 kg/m^2^, he was obese. A neurological test indicated a power of 1/5 and a markedly lower tone in each of the four limbs. There was a reduced reaction in the lower and upper limbs and an inconclusive plantar reflex. To rule out any intracerebral pathology, a plain CT scan of the brain was performed and was found to be normal. Upon presentation, it was discovered that he had severe hypokalemia, with potassium levels of 1.9 mEq/L along with thrombocytopenia, with platelet counts of 40,000. Urinary potassium loss was ruled out when the urine's creatinine/potassium value was examined and found normal. The levels of magnesium were likewise normal. Immediately after, intravenous potassium chloride infusion via a central line was started to correct potassium in accordance with general care of hypokalemia guidelines. The potassium level was checked every 12 hours and gradually increased. Over the course of 48 hours, we replaced 240 mEq of potassium, and readings were normalized to 4.1 (Table 1).

**Table 1:** Blood investigations g/dl: grams per deciliter; mg/dl: milligrams per deciliter; mmol/L: millimoles per liter;U/L: Units per liter; CRP: C-reactive protein; AST: aspartate aminotransferase; ALT: alanine transaminase

Date	Hemoglobin	Packed cell volume	Platelets	Creatinine	Sodium	Potassium	CRP	AST	ALT	ALP	GGT
5/11/22	9.6	30.5	0.9	0.8	136	2	37	70	-	141	58
5/11/22	10.1	31.8	0.4	-	-	2.2	46.7	-	-	-	-
6/11/22	10.6	33.2	0.19	-	-	2.4	-	-	-	-	-
7/11/22	-	-	0.2	-	-	4	-	1415	-	-	-
8/11/22	10.7	33.3	0.22	-	131	4.1	82.6	-	-	-	-
9/11/22	9.6	30.2	0.68	-	-	4.2	-	914	456	-	-
10/11/22	9.9	31.5	0.6	0.8	134	4.5	79	97	79	247	289
Reference range	14-18 g/dl	42-54 %	1.5-4.5 lakhs/microliter	0.42-1.06 mg/dl	135-145 mmol/L	3.5-5.1 mmol/L	< 5 mg/dl	0-40 U/L	0-40 U/L	30-130 U/L	0-40 U/L

The patient's four limbs gradually acquired strength. After a nerve conduction velocity and electromyography, all four limbs were found to be normal. Due to dengue, his platelet counts were also regularly checked, and on two occasions, a platelet transfusion was necessary. Hyperinsulinemia was suspected as part of the examination of hypokalemia. His glycosylated hemoglobin (HbA1C) was within the prediabetic range at 6.3. Given his obesity, we sent his C peptide level, which came back at 8.52 (1.10-4.40), which was expectedly high. A higher creatinine phosphokinase suggested the possibility of myositis, which subsequently returned to normal after hydration. The patient was sent home without any complaints after he became afebrile and could move around without assistance. In light of the patient's hyperinsulinemia, he was diagnosed with hypokalemic periodic quadriparesis induced by dengue fever.

## Discussion

The link between obesity and insulin resistance is widely accepted today and was described by Kahn et al [5]. Dengue frequently causes myositis and muscle involvement. Thirty-one out of the 39 dengue patients (more than half) had clinical symptoms, according to research by Mishra et al. showing indications of clinical muscle involvement [6]. Although our patient's most recent potassium readings were unavailable, it was plausible to presume that his hypokalemia was acute given the abrupt onset of his muscle weakness. By boosting the activity of the Na K ATPase pump, elevated insulin facilitates the entrance of potassium into skeletal muscle and hepatic cells. Therefore, the effect of insulin on blood potassium levels will be the same even in cases when the insulin is endogenous. Upon assessing the reason behind the elevated insulin levels in this patient who was not yet diabetic, no evident pathology, such as insulinoma, or endocrine cancer, was discovered.

Insulin resistance development is linked to obesity in the pre-diabetic state [6]. In the presence of dengue viral infection, the patient's hyperinsulinemic state, which is demonstrated by his pre-diabetic range of HbA1c (6.1), obesity, and elevated C-peptide values, may have been the cause of the acute hypokalemic state. The stress of the virus might have triggered more insulin release, which would have exacerbated the hypokalemia. Insulin resistance and downregulation of IRS 1 are caused by a dengue virus infection, as shown by Liu et al. [7]. In a case report published by Akthar et al., a 30-year-old patient with lower limb paralysis and fever was later confirmed to have positive dengue serology [8]. A comparable case with a 26-week pregnant woman who suddenly developed limb weakness and a high-grade fever was reported by Khan et al. [9]. In a retrospective analysis of 29 individuals with hypokalemic paralysis, Garg et al. discovered that four (13.7%) of them had dengue [10]. Although there have been a number of cases reported that are similar, none that examine the connection between this phenomenon and a hyperinsulinemic state were found. Jha and Ansari suggest that there are two potential explanations for dengue hypokalemia: either potassium is redistributed into the cells, or there are temporary renal tubular abnormalities that result in an increased excretion of potassium in the urine [1].

Since the potassium/creatinine ratio in the urine was normal, the main cause of the hypokalemia in this instance was likely an intracellular redistribution of potassium. A further source of identical dengue signs, Guillain-Barre syndrome, was ruled out by normal nerve conduction studies and electromyography. It's interesting to notice that the myositis and hypokalemia developed nine days after the fever initially started, during the dengue critical phase. Since this entity has been mentioned in several case reports from India, it is crucial to maintain a high degree of skepticism about it after the dengue fever has subsided [11-13].

## Conclusions

It is crucial that all medical professionals, particularly those working in tropical regions, are aware of this entity and can identify it at an early stage. Even while severe and abrupt hypokalemia is not typically associated with dengue, this case emphasizes how important it is to have a high suspicion of this condition in dengue patients. Dengue fever should be investigated in any patient who presents with limb paresis and thrombocytopenia, even if the patient has not had a fever in the last week or has not had one in the past. This includes our patient. This case study is one of many that have been released in the past 10 years, most of which are from the subcontinent. It is generally acknowledged that obesity and dengue virus infection are linked to insulin resistance and hyperinsulinemia. Obesity and dengue virus infection may have both led to elevated insulin production, which in turn caused our patient's severe hypokalemia. To investigate the evidence of the same, we need to conduct additional research on this patient group.

## References

[1] Jha S, Ansari MK (2010). Dengue infection causing acute hypokalemic quadriparesis. Neurol India.

[2] Roy A, Tripathi AK, Verma SP, Reddy H, Jain N (2011). Acute hypokalaemic quadriparesis in dengue fever. BMJ Case Rep.

[3] Gupta DK, Vaish AK, Arya RK, Chaudhary SC (2011). Hypokalaemic quadriparesis: an unusual manifestation of dengue fever. BMJ Case Rep.

[4] Johnkennedy N, Chibuzor OP, Chidozie NJ (2022). Perspective of channelopathy in diabetes mellitus. Biomed J Scientific Tech Res.

[5] Kahn BB, Flier JS (2000). Obesity and insulin resistance. J Clin Invest.

[6] Misra UK, Kalita J, Maurya PK, Kumar P, Shankar SK, Mahadevan A (2012). Dengue-associated transient muscle dysfunction: clinical, electromyography and histopathological changes. Infection.

[7] Liu X, Liang Z, Duan H (2022). Dengue virus is involved in insulin resistance via the downregulation of IRS-1 by inducing TNF-α secretion. Biochim Biophys Acta Mol Basis Dis.

[8] Akhtar U, Mushtaq MH, Nisar S, Naeem A, Naim F (2023). Dengue and hypokalemic paralysis: a rare association. Cureus.

[9] Khan AB, Ali MA, Ur Rehman S, Siddiqe U, Ahmad S (2023). Dengue fever-induced hypokalemic paralysis in a pregnant patient: an uncommon presentation of a common disease. Cureus.

[10] Garg RK, Malhotra HS, Verma R, Sharma P, Singh MK (2013). Etiological spectrum of hypokalemic paralysis: a retrospective analysis of 29 patients. Ann Indian Acad Neurol.

[11] Kaur M, Singh P (2021). Acute dengue myositis: a case report. AMEI’s Curr Trends Diagn Treat.

[12] Paliwal VK, Garg RK, Juyal R (2011). Acute dengue virus myositis: a report of seven patients of varying clinical severity including two cases with severe fulminant myositis. J Neurol Sci.

[13] Sadiqa A, Hayyan U, Waqar Z, Pasha T (2023). Myositis as a complication of dengue viral infection. Pak J Med Sci.

